# Informed Adaptations of a Strength-Training Program through a Research–Practice Partnership

**DOI:** 10.3389/fpubh.2018.00058

**Published:** 2018-03-02

**Authors:** Meghan L. Wilson, Thomas E. Strayer, Rebecca Davis, Samantha M. Harden

**Affiliations:** ^1^Department of Human Nutrition, Foods, and Exercise, Blacksburg, VA, United States; ^2^Translational Biology, Medicine, and Health, Blacksburg, VA, United States; ^3^Virginia Cooperative Extension, Virginia Tech, Blacksburg, VA, United States

**Keywords:** integrated research–practice partnership, adaptations, implementation, strength-training, older adults

## Abstract

Efficacy and effectiveness data for strength-training programs targeting older adults have been well established, but it is evident that they are not translated within practice-based settings to have a public health impact, as most (~90%) older adults are not meeting strength-training recommendations. Strength-training interventions developed, delivered, and evaluated in highly controlled settings (e.g., eligibility requirements, certified instructor, etc.) may not reflect real-world needs. One strategy to improve these outcomes is to work through an integrated research–practice partnership (IRPP) to plan and evaluate an intervention to better fit within the intended delivery system. The purpose of this study was to describe the IRPP method by which academic and practice representatives can partner to select and adapt a best-fit strength-training program for older adults. This work was planned and evaluated using the reach, effectiveness, adoption, implementation, and maintenance framework, applying the AIM dimensions to complement the methodology of the partnership. In this pragmatic work, members of the IRPP adapted the evidence-based program, Stay Strong, Stay Healthy (SSSH) into a new program, Lifelong Improvements through Fitness Together (LIFT). Of the health educators who agreed to be randomized to deliver LIFT or SSSH (*N* = 9), five were randomized to SSSH and four were randomized to deliver LIFT. Fifty percent of educators randomized to SSSH delivered the program, whereas 80% of the health educators randomized to LIFT delivered the program. The health educators deemed LIFT more suitable for delivery than SSSH, self-reported high rates of fidelity in program delivery, and intended on delivering the program in the following year. In conclusion, this study provides transparent methods for using an IRPP to adapt an intervention as well as preliminary outcomes related to adoption, implementation, and maintenance.

## Introduction

A number of strength-training programs for older adults that include home-based resistance training, supervised and unsupervised strength training, and strength training with groups of individuals have been effective (1–3). Unfortunately, less than 10% of the older adult population are meeting strength-training recommendations (4). This indicates that while the efficacy for these programs is well established, they are not being translated within practice settings to have a public health impact.

Many evidence-based programs restrict their eligibility requirements based on medical status such as having osteoarthritis, living with diabetes, or those who identify with a specific gender (5–7). Similarly, some programs require delivery agents to be trained or certified fitness instructors through an external affiliation (i.e., American College of Sports Medicine) (8). These highly controlled conditions (e.g., eligibility restrictions for participants, certification requirements for delivery personnel, etc.) focus primarily on internal validity under best practices and may impede successful intervention implementation from one setting to the next due to limited attention on external validity (9–11).

Limiting the “fit” of an intervention leads to the absence of full penetration within a delivery setting and potential reach of the priority population, a shortcoming of delivery agents willing to adopt the intervention, and ultimately, inadequate implementation of the intervention (12, 13). To improve the potential for an intervention to be translated in the intended delivery system, interventions should be developed considering both internal and external validity factors (e.g., will the intervention work for multiple subgroups of populations in various settings and will it have greater advantage over alternative interventions?) (11).

One way to systematically capture these factors is to plan and evaluate a strength-training intervention using the RE-AIM (reach, effectiveness, adoption, implementation, and maintenance) framework (14). Accurate reporting of these components may lead to the development of an intervention that will have long-term, practical application and pragmatic fit within the practice-based setting. There remains a publication bias toward effectiveness outcomes. Intervening to improve adoption, implementation, and maintenance outcomes is nascent for strength-training promotion interventions for older adults (11, 15).

To improve adoption, implementation, and maintenance outcomes of behavior change interventions, interventions should identify and align with the existing resources, mission, and values of practice-based settings and staff (e.g., lay-, peer-, or professional-health educators) (16, 17). Health educators have the expertise, access, and willingness to deliver community- and evidence-based programs within their setting if they are easily translatable and sustainable in practice (13, 18, 19). The implementation strategies used to adopt and integrate evidence-based health interventions into specific settings, should be deemed acceptable, appropriate, and feasible for those delivering and receiving the intervention in a specific clinical or community setting (9, 20). For example, an intervention will not be delivered with fidelity if it does not “fit” the mission and values of the individual- or setting-level delivery system or the priority population, resulting in compromised effectiveness of the intervention (18, 21–23).

One system with a particular mission to adopt and deliver effective behavior change interventions is the Cooperative Extension (CE) Extension system. Extension health educators represent communities and may serve as appropriate facilitators of behavior change interventions related to strength training because of their preexisting relationships with community members and leaders, access to community resources, and understanding of their communities needs (24). However, health educators are often compelled to deliver various programs addressing the needs of varying priority populations (adolescents, adults, seniors, etc.) based on an annual situational analysis conducted in their counties (18). Selection and delivery of programs is often based on personal experience, observability, or a top down dissemination approach where the academic researcher produces a program that the health educator will deliver (25). This selection and delivery approach disregards specific community needs, values, and ability, which may lead to poor translation of effective, evidence-based strength training programs into a practice-based setting (25). Furthermore, the process by which these health educators can work with researchers to swiftly and effectively select and adapt an intervention has yet to be reported in the literature.

Through an integrated research–practice partnership (IRPP), health educators and researchers equally contribute toward identifying a public health concern, design an intervention that is feasible, acceptable, and appropriate for delivery within the delivery system, and transparently report lessons learned to advance the utility and translation of the intervention within the intended delivery system (9, 26, 27). As a result, health educators may perceive a program as more suitable and acceptable for delivery if it has been collaboratively adapted in accordance with the underlying program principles that incorporates the ideas and needs of those delivering and receiving the program (9, 28–30). Establishing an IRPP between academic and community partners may bridge the gap of research-based knowledge to practice-based expertise for accelerated implementation and dissemination of evidence-based, effective interventions into real-world, pragmatic settings (31, 32).

The overall purpose of this article is to describe the process by which an evidence-based, strength training program underwent research- and practice-based adaptations through the scope of a partnership to develop a program that would be adopted by community health educators, implemented throughout the community with fidelity, and maintained in a practice-based setting, beyond the life of the intervention (31, 33). Described within is the process of incorporating, reporting, and testing the feasibility or fit of evidence- and practice-based adaptations into a strength-training intervention based on the needs of the health educators and the priority population.

## Method

### Integrated Research–Practice Partnership

In order to introduce quality programs that focus on physical activity within Virginia’s Extension system and improve older adult individual-level strength-training compliance, an IRPP was formed among health educators of Extension and behavioral and implementation scientists in 2015. Specifically, the partnership consisted of nine health educators across each of the four districts of Virginia Extension who had previously delivered physical activity programs to their community and one exercise specialist (statewide exercise leader of Extension). The partnership also included three graduate research assistants and two external health educators with communities in need of older adult physical activity programing. The stakeholders involved in the partnership labeled themselves as the Physical Activity Leadership Team. Members of the Physical Activity Leadership Team agreed to meet biannually in-person at a central location and biannually *via* WebEx for regular check-ins, program updates, and adaptations needed by members to better suit their needs for delivery of physical activity programs. All study procedures were completed in accordance with and with the approval of the Virginia Tech Institutional Review Board, participation was voluntary, and all data were kept confidential. All participants gave written informed consent before participation.

### Intervention Identification, Selection, and Structure

Based on the research- and practice-based evidence of effective and sustainable strength-training programs for older adults in Extension, Stay Strong Stay Healthy (SSSH) (34) and Better Bones and Balance (35) were presented to the members of the partnership within Virginia Extension. Based on the needs and constraints of Virginia Extension health educators, SSSH was the program of choice by the Physical Activity Leadership Team.

**Figure 1:**
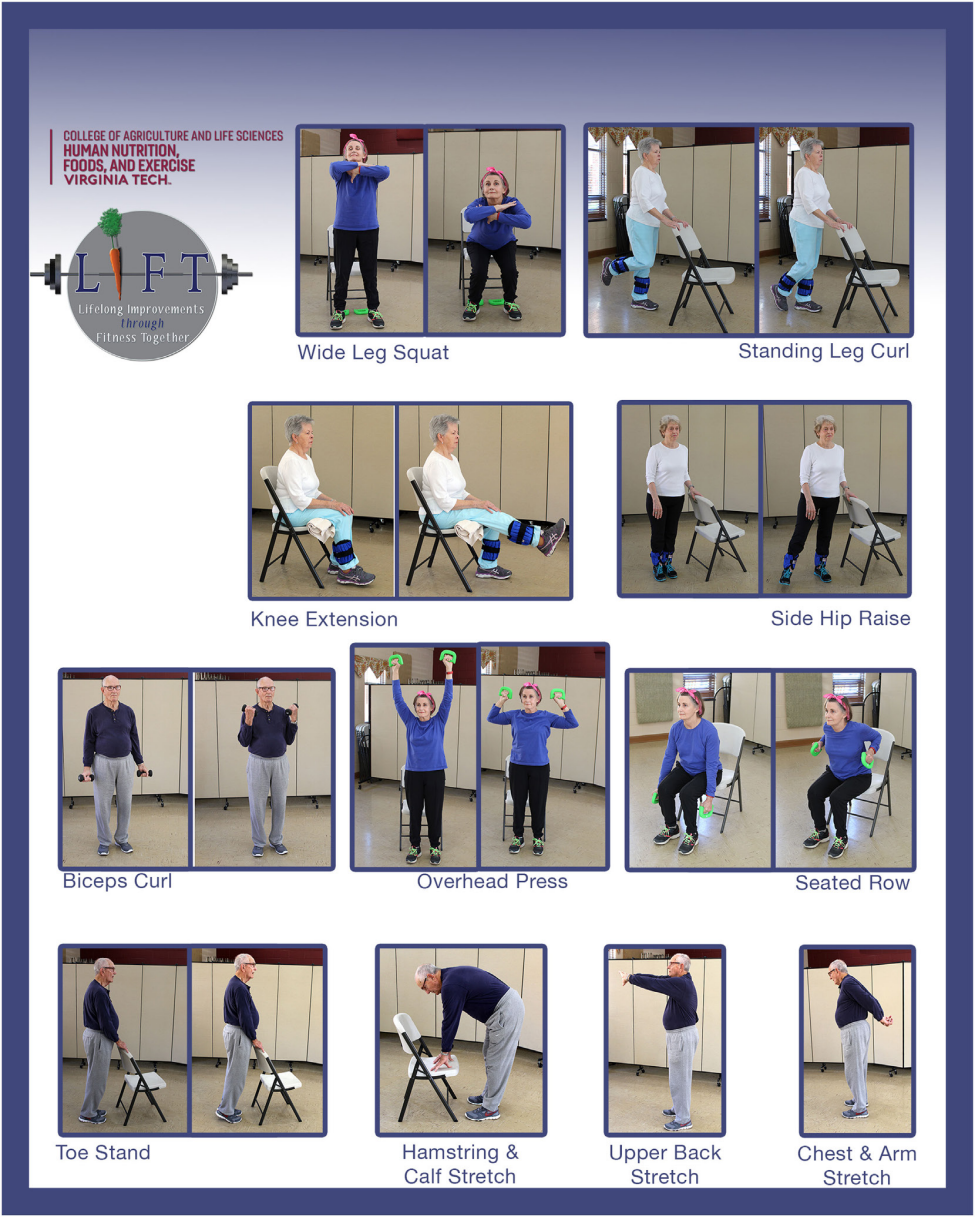
Strength-training exercises and cool-down stretches of Lifelong Improvements through Fitness Together (LIFT) and Stay Strong Stay Healthy.

Each SSSH session is intended to last 1 h and consists of strength-training, balance, and flexibility exercises, and cool-down stretches (Figure 1). Functional fitness measures are collected and recorded before and after completion of the program to assess intervention effectiveness. Participants are encouraged to attend one to two sessions per week for 10 weeks but no behavioral change strategies are incorporated into the sessions (i.e., goal setting, self-monitoring, group distinction, etc.). To summarize, the core elements of SSSH are 10–20 in person sessions, a warm-up, 8 strength training exercises, and 3 cool down stretches (34). The underlying core components of SSSH can be seen in Table 1.

**Table 1:** Underlying core components of LIFT versus SSSH.

Core component	SSSH	LIFT
Duration	1 h sessions, 1–2 times a week for 8 weeks	1 h sessions, 2 times a week for 8 weeks
Audience	Insufficiently active, aging men and women	Insufficiently active, aging men and women
Behavioral components	No strategies used	Observational learning, self-monitoring, self efficacy, group dynamics, and relapse prevention
Exercises	Active warm-up, 8 core strength-training exercises, and cool down	Active warm-up, 8 core strength-training exercises, and cool down
Group dynamics	No strategies used	Small groups for interaction, group names; participant lead exercises; friendly competition, social support, group goals
Goal	Increase muscle and bone density to decrease osteoporosis and frailty	Enhance overall functional fitness through improved strength, flexibility, and balance

**Figure 2:**
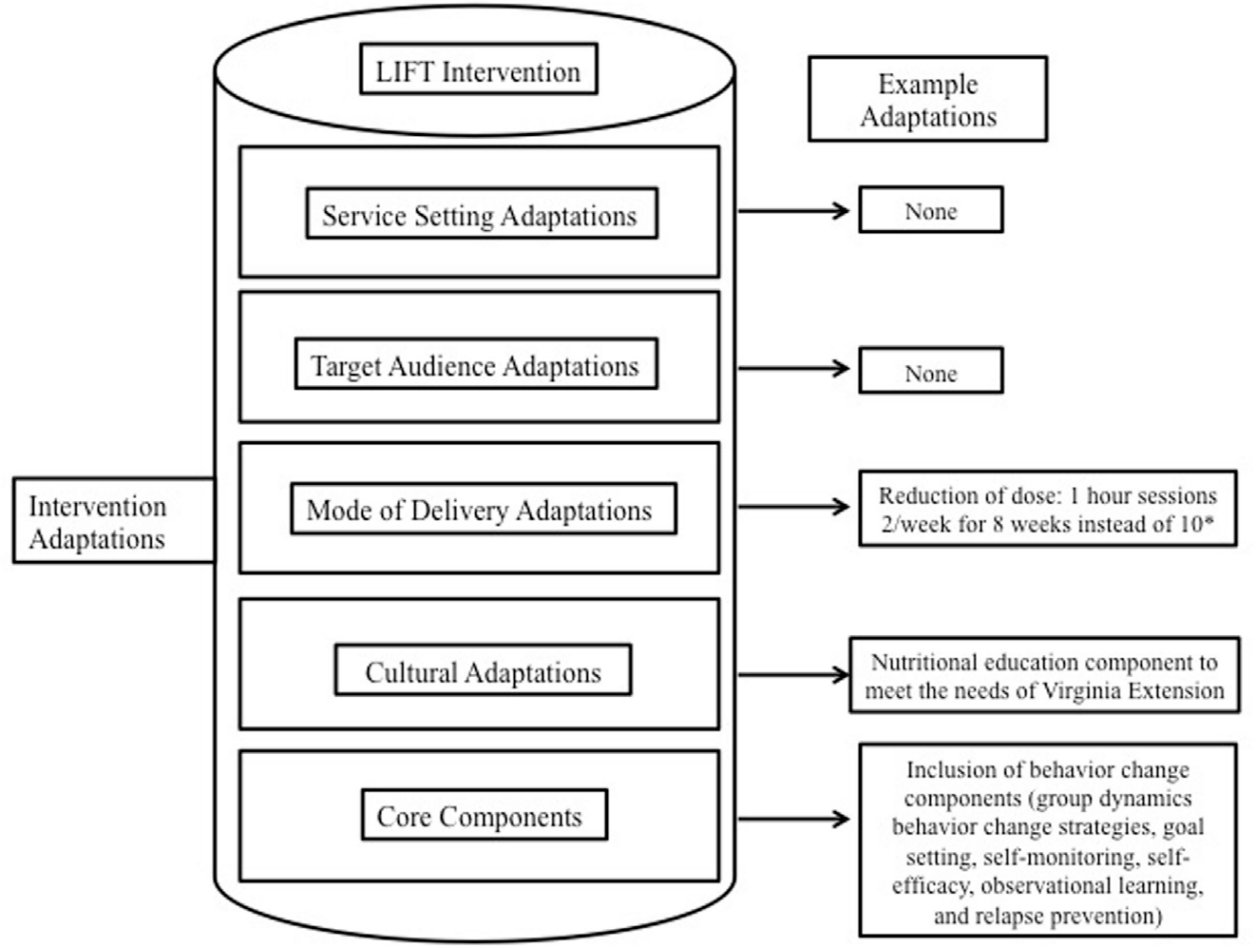
Evidence- and practice-based adaptations of Lifelong Improvements through Fitness Together (LIFT). This figure is adapted from Chambers and Norton (44). *SSSH was also adapted from a 10-week program to an 8-week program; however, those results are not yet published.

However, the Physical Activity Leadership Team requested evidence- and practice-based adaptations before translating SSSH into the Virginia Extension system. These adaptations (Figure 2) included (1) the facilitation of group dynamics-based behavior change strategies; (2) a nutrition education component embedded within the program to align with the Virginia Extension mission; (3) physical activity and fruit and vegetable consumption tracking [i.e., self-monitoring (36)]; and (4) the reduction of in-person time commitment (delivery of the program for 8 versus 10 weeks). The newly adapted program implemented across the state of Virginia is called Lifelong Improvements through Fitness Together (LIFT).

### Adaptations to Intervention

#### Group Dynamics

To translate SSSH into Virginia Extension system, the exercise specialist of the Missouri Extension system delivered an in-person training on SSSH to the exercise specialist and one health educator of the Virginia Extension. While at the training, Missouri health educators that have delivered SSSH to aggregates of individuals, anecdotally noted that “camaraderie” was a by-product of participation in SSSH. However, camaraderie is not a “natural” by-product of working out among a group of individuals. For example, anyone can attend a weekly group fitness exercise class without actively engaging or communicating with the people around them. Implementing a group dynamics-based approach in which communication and interaction is facilitated between participants within a setting can foster group cohesion (37) or, “the dynamic process reflected by the tendency of a group to stick together and remain united in the pursuit of its instrumental objectives and/or for the satisfaction of member affective needs” (38).

A group dynamics-based intervention is one approach that has been successful with older adults (39). This approach includes the active facilitation of strategies (e.g., goal-setting, friendly competition, group distinction, etc.) to promote a greater perception of group cohesion (37). The groups’ cohesiveness should be actively facilitated or targeted through group dynamics-based strategies to enhance the feeling of belonging and encourage communication among group members (40) over the course of the intervention. Health educators delivering LIFT were trained on how to identify and target the underlying group dynamics-based principles (goal-setting, group distinction, roles, friendly competition, etc.). Prior to delivering LIFT, health educators were provided with strategies to satisfy the underlying group dynamics-based principle of the session (e.g., encouraging members to come up with a team name), but could adapt the strategies to meet the needs of their priority population (e.g., instead of identifying with a group name they could all wear similar colored shirts). Health educators understood the importance of transparently reporting the planned change or adaptation made from the strategy provided.

#### Nutrition Education

Although most Extension programs focus on nutrition education and integrate components of physical activity (41), LIFT is an exercise program that incorporates nutrition education. This was done to meet the mission and values of Virginia Extension. Each LIFT session included a short nutritional education piece concerning the importance of increased fruit and vegetable consumption. Delivery agents were able to present the provided educational information to their participants in concurrence with the in-person LIFT session.

#### Self-Monitoring Behavior

To influence motivation toward long-term behavior change, participants are encouraged to track their physical activity behaviors and fruit and vegetable consumption outside of the LIFT program. Participants were provided with a self-tracking sheet that they could complete at their own leisure. Self-tracking and self-monitoring of behavior helps individuals regulate their behavior and stay motivated toward long-term behavior change (36).

#### Dose

Reported results of SSSH inform that health educators deliver the intervention twice a week for 10 weeks (34). SSSH has recently been adapted to be delivered twice a week for 8 weeks; however, those results are not yet published. To align with the current (however not published) dose of SSSH within Missouri Extension Virginia Extension delivered LIFT twice a week for 8 weeks. This ensures that participants are engaged in adequate strength training to meet the physical activity guidelines, but is not burdensome to those delivering or receiving the intervention.

To summarize, the core components of LIFT are 16 in person sessions, the same warm-up, strength-training exercises, and cool down stretches from SSSH (34), and the inclusion of behavior change strategies that fit the mission and values of Virginia Extension, the health educators delivering the intervention, and older adults receiving the intervention (Table 1: Underlying core components of LIFT versus SSSH comparison).

### Recruitment and Training

In 2015, all Masters trained and professional Virginia Extension health educators (*N* = 52) were invited to attend a 2-day physical activity training hosted by the lead and senior authors. Thirty-four Virginia Extension agents signed up to attend. Over the course of 2 days (5 h on day 1 and 6 h on day 2) all agents learned the following: (1) the primary evidence-based principles of reaching physical activity behavior change, (2) strength-training exercises (proper form, cues to deliver, safe speed, etc.), (3) the importance of evaluation tools (surveys, functional fitness assessment, and demographic information), and (4) personal experience and practice in leading groups through strength-training exercises. Specific to LIFT, health educators received a thorough information session, *via* PowerPoint Presentation, introducing the evidence-based strategies and specifics of the program. The information session was followed by a 2-h demonstration consisting of the eight, full-body exercises, functional fitness assessments, and a testimonial from the perspective of a health educator who has delivered physical activity interventions within communities she serves for over 10 years. Health educators also spent 2 h of experiential learning and performing a sample LIFT session (training materials available upon request).

Before a full launch of LIFT across the state of Virginia, it was essential to test the feasibility of the adaptations and build the evidence-base of the program. Health educators interested in participating in a feasibility trial (*n* = 13) of LIFT versus SSSH were invited to attend a 3-h, in-person information session where they were (1) refamiliarized with the adapted program, LIFT and (2) given repeat demonstrations of the eight strength-training exercises and functional fitness assessments in January 2016. Eligible attendees included health educators who either (a) attended the training in 2015 or (b) were members of the Physical Activity Leadership Team. The purpose of the feasibility study was to evaluate the potential reach of LIFT and practicality of implementing program adaptations for sustainable delivery within the Virginia Extension system, ensure that the evidence- and practice-base adaptations did not compromise the effectiveness of the intervention, and determine health educator level-adoption, and maintenance, or intentions of continued delivery in a community-based setting.

## Measures

### RE-AIM

*R*each and intervention *e*ffectiveness data were collected from pre- and post-program surveys and functional fitness assessment for the feasibility study. However, those data are part of a larger trial and are not reported within this manuscript. *A*doption data were collected as the proportion of eligible and interested health educators who agreed to deliver LIFT or SSSH as part of the feasibility study for assessment. Health educator participants also completed weekly process evaluations (see Table S1 in Supplementary Material, for an example process evaluation) to capture the *i*mplementation and feasibility of current adaptations and further report real-world adaptations made during class sessions. Although health educators were free to change the suggested activities of the program, adaptations were still recorded to ensure they did not deviate from the underlying core components of LIFT and to gather a repository of adaptations that captures for whom, what, how, and when (42) adaptations were deemed necessary by health educators for their priority population. The process evaluations (see Table S1 in Supplementary Material, for an example process evaluation) also informed the partnership of self-report program fidelity, or the extent to which the program was delivered as intended in regards to the posed adaptations made in alliance with each activity, with results reported in five activity categories (e.g., warm-up activity, group-dynamics strategy, exercises, cool down, and overall program delivery). Proportions of program fidelity were calculated based on self-reported delivery adherence by activity, when compared to the total number of sessions delivered. Implementation fidelity was deemed appropriate if changes made during the LIFT program did not deviate from the underlying core principles (group dynamics-based strategies, exercises, dose, frequency, etc.). *M*aintenance was operationalized as the health educators’ intent to deliver (yes = intend to deliver; no = do not intend to deliver) the program in the future.

## Results

### Adoption

Of the 13 eligible and interested health educators who expressed the need for translating LIFT into their community, 9 (70%) agreed to deliver LIFT or SSSH as part of a feasibility trial. Five of the nine health educators were randomized to LIFT and four delivered the program to older adults in the community. Four of the nine health educators who agreed to participate in the feasibility trial were randomized to SSSH and two delivered the program to older adults in the community.

Using the denominator of 9 (those eligible and agreed to deliver), 4/9 (44%) delivered the LIFT program in practice, whereas 2/9 (22% of those eligible and agreed to deliver) delivered the SSSH program in practice. However, using the denominator of 13 health educators, i.e., total number eligible and *interested* in delivering the program, the proportion delivering LIFT was 4/13 (31%) whereas the proportion delivering SSSH was 2/13 (15%).

### Implementation

#### Warm-Up

Health educators reported on delivering the program with high fidelity 58% of the time for warm-up exercises. When a health educator reported an adaptation during the warm-up activity, the adaptation included, “completed high knees and grapevine exercises instead of marching in place while pumping arms.”

#### Group Dynamics-based Activities

Health educators reported high fidelity to the group dynamics-based activities 64% of the time. However, when an adaptation was reported, they stated within the adaptation that the activity did not fit their priority audience or there was not sufficient time within the session to complete the activity. An example adaptation reported was, “Instead of encouraging everyone in the group to perform the specific activity, I would ask for volunteers to complete the activity to save on time.”

#### Exercises

Health educators reported doing more repetitions of exercises than was prescribed in the program manual 38% of the time. Only one health educator reported doing less than the number of repetitions prescribed (i.e., her participants did not complete a second set of arm curls and overhead press exercises throughout the duration of the LIFT however, they increased the number of repetitions in the first set to 12). Health educators reported changes in the equipment used to complete exercises 10% of the time. With regard to equipment changes, an example adaptation included, “used resistance bands in place of dumbbell weights.”

#### Cool-down

Health educators reported delivering the cool down stretches as intended 89% of the time. When a change in exercise was made, health educators reported the adaptation as, “participants wanted to walk around the gym instead of stretch” or “completed different stretches.”

#### Overall program delivery

Health educators reported delivering the program as intended 99% of the time, overall.

### Maintenance

One hundred percent of the health educators who delivered LIFT reported “yes” that they had intentions to deliver LIFT in the future. Of those who delivered SSSH, 100% confirmed their intentions (yes) to deliver LIFT in the future instead of SSSH. The health educators who delivered SSSH expressed their desire to incorporate the nutrition component and active facilitation of the social environment for their older adult participants. To date, five of the six health educators have continued delivery of LIFT within their community to different cohorts of older adults. The health educator not delivering LIFT has since left Virginia Extension.

## Discussion

The overall purpose of this article was to describe the process by which an evidence-based intervention underwent research- and practice-based adaptations through an IRPP approach. Results of preliminary fit of the adapted intervention were based on the Adoption, Implementation, and Maintenance dimensions of the RE-AIM framework. Together, the IRPP identified an evidence-based program, adapted it to meet the mission and values of the Extension setting and staff, delivered the intervention, and transparently reported any further, real-world adaptations made during delivery.

Lifelong Improvements through Fitness Together, the adapted intervention of SSSH, was delivered in practice at a higher rate than the original program, although the sample size was too low to determine significance (Adoption). Although SSSH continues to show positive effects in strength-training behavior and has been delivered and sustained within the Missouri Extension system for over 10 years, the adapted intervention, LIFT, was deemed more appropriate than SSSH within the intended setting for the priority population. This is unsurprising as LIFT was adapted to fit the needs, values, and resources of the existing delivery system (Implementation) (12, 34). Working through an IRPP built buy-in from stakeholders that helped facilitate their desire to understand and capacity to employ the underlying program principles that aligned with the proposed adaptations (i.e., group dynamics, self-monitoring, and dose) and deliver the program with a high degree of fidelity to obtain intervention effectiveness (12, 30, 43). Continued efforts of the IRPP to promote the “appropriateness” of LIFT for older adults across the state of Virginia may improve the adoption, implementation, and maintenance rate of LIFT and may have a greater effect on the strength-training behaviors of older adults across the state.

Evidence-and practice-based adaptations are planned and executed to improve the feasibility, acceptability, appropriateness, and overall fit and delivery of an intervention in a given system. Adaptations are not to be mistaken for program deviations or unplanned changes made throughout an intervention that have a negative impact on system-level or participant-level outcomes (21, 44). The process described in this manuscript aimed to contribute to the literature identifying what, when, and by whom adaptations were made. Communicating to members of the IRPP the importance and utility of the information provided with transparent reporting of adaptations made to the program manual is a key component to improving the potential for satisfactory intervention fidelity (45). However, the fidelity data specific to this intervention were difficult to interpret for a number of reasons. First, to the authors’ knowledge, there was not an existing, psychometrically tested fidelity checklist for these interventions. The fidelity rate should be interpreted with caution. Second, the fidelity data were collected as self-report perceptions of delivery, meaning, that although they were reporting changes to the activities, they were still achieving the goal of the program (e.g., improve strength of older adults, engage in social activities to build cohesion, etc.). Findings were brought to the health educators of the IRPP and found that, although the data related to individual components of the program (warm-up, group dynamics activities, exercises, cool down, and overall program delivery) did not indicate that they were delivered with 100% fidelity, health educators believed, or reported high fidelity that the overall program was delivered as intended based on the adaptations made.

It can be concluded that although health educators were reporting adaptations being made or changing the “form” of the warm-ups, group dynamics-based activities, exercises, or cool downs, those adaptations were not changing the “function” of the activity. Meaning, the adaptations being made were still targeting the underlying program principles and core components of LIFT (46). It is important to note that “manualized” interventions are nearly impossible to ensure across multiple, pragmatic settings and that adaptations should be encouraged if they (1) align with the underlying program principles, (2) can be broadly delivered by delivery agents, and (3) best meet the needs of the participants (44). Future work is needed to develop, test, and validate a psychometric process evaluation to more effectively gather fidelity data for this type of intervention.

Providing health educators who will deliver LIFT in the future with a repository of changeable strategies to target the underlying core components of LIFT, will allow them to better meet the individual needs of their participants (47) and may further increase program feasibility for delivery personnel within a setting (9). This strategy enables health educators to tailor the intervention to improve the overall appropriateness, acceptability, and fit across a wide variety of settings for participants with varying needs (i.e., senior centers with individuals in wheelchairs, clinics with patients at risk for falling, hospitals with patients diagnosed with chronic illnesses or mobility restrictions, etc.) without compromising the effectiveness of the intervention (9, 44). Having a centralized resource that includes the types of adaptations made, for whom they were made for, and at what point in the intervention they were made will better inform the adoption and implementation decisions of health educators interested in delivering LIFT (42).

Inevitably, real-world adaptations often occur in all practice settings and implementation with satisfactory fidelity to the intervention design is rarely achievable (22, 44). Through an IRPP approach, research team members were able to conclude that the adaptations made in practice and reported on within the process evaluations (see Table S1 in Supplementary Material, for an example process evaluation) adhered to the underlying program principles of LIFT. This indicates four things: (1) the process evaluation approach (and analysis) will be amended to better reflect the degree to which the program aligns with research-based principles, (2) although the process evaluations are self-report, the intervention effects were not compromised (effectiveness data were collected as part of a larger trial, reported elsewhere) and therefore the process evaluations may provide support for relative intervention adherence or satisfactory fidelity, (3) health educators are able to make pragmatic adaptations that adhere to underlying program principles, and (4) through the use of process evaluations we were able to highlight how to better report the process of real-world adaptations occurring and to better understand how the adaptations impact those at the individual level in different settings (18, 25, 44, 48).

There were few limitations to be addressed. First, research members of the partnership stressed the importance of transparent communication between all members of the partnership and felt it would be unfair to blindly randomize members to deliver LIFT or SSSH. Instead, there was open communication about the importance of testing the feasibility of the two interventions within the Virginia Extension system and further build the evidence-base for LIFT within Virginia. However, once each program was assigned to the respective communities, health educators expressed disappointment in being assigned to the SSSH protocol, as they wanted to incorporate behavior change strategies and actively interact with their study participants. The research team further elucidated the importance of the feasibility trial, however, there may be program drift that went undetected due to the small sample size (44). Second, the research team used a convenience sample of health educators due to their excitement and willingness to deliver a strength-training program to older adults within their communities. However, we understand the barriers of delivering physical activity interventions, specifically strength-training, and future research will be needed to target health educators with lower self-efficacy for delivering such programs. Lastly, the results of this trial are only generalizable to the health educators of Virginia however; recruitment of health educators across various counties within Virginia allowed the research team to analyze the results from multiple regions and different settings across the state. To date, the partnership is utilizing external relationships to expand the delivery of LIFT across multiple states.

## Conclusion

The research–practice partnership implementation strategy allowed the research team to work with the intended delivery system and the health educators within the system to improve the quality of the intervention integrated within the setting. Although this work was conducted in Extension, the method can be applied in other pragmatic settings. Through results of this study, it is evident that working through a research–practice partnership and involving stakeholders in the design and delivery of an intervention is key in the successful implementation of an intervention. This approach allowed for real-time, transparent tracking of further adaptations needed by the health educators and intervention participants, did not compromise the effectiveness of the program for the priority population, and resulted in relatively successful program fidelity.

Together, researchers and invested stakeholders can identify a need that targets a specific community, design or tailor an existing intervention to improve the overall fit within the community, test the implementation outcomes (feasibility, acceptability, appropriateness, sustainability, etc.), and determine if the intervention is ready for full-scale translation into a specific setting. The process of implementing an intervention into a specific setting should be nonlinear and simultaneous, meaning that the IRPP should design, test, and deliver the intervention while planning for long-term sustainability from the beginning.

## Ethics Statement

All study procedures were completed in accordance with and with the approval of the Virginia Tech Institutional Review Board, participation was voluntary, and all data were kept confidential. All participants gave written informed consent before participation.

## Author Contributions

MW: significant contribution toward writing and editing the manuscript. Also collected, analyzed, and reported results of data. TS: significant contribution toward editing the manuscript and overseeing data collection, analyzes, and reports. RD: significant contribution toward editing the manuscript. Was also our program champion within our research–practice partnership. Helped to spear head meetings, trainings, program delivery, data collection, etc. SH: Significant contribution toward writing and editing the manuscript. Oversaw all research activities.

## Conflict of Interest Statement

The authors do not have any conflicts of interest to declare and results of this study do not constitute endorsement by the American College of Sports Medicine.

## Abbreviations

LIFT, Lifelong Improvements through Fitness Together; SSSH, Stay Strong Stay Healthy.
