# Supplementary material for: Informed Adaptations of a Strength-Training Program through a Research–Practice Partnership
Source: Front Public Health. 2018 Mar 2;6:58. doi: 10.3389/fpubh.2018.00058 (PMC5840863; doi:10.3389/fpubh.2018.00058)
Supplement: Supplementary file 1 [file Table_1.docx]

Supplementary Material

**Informed adaptations of a strength-training program through a research-practice partnership**

Meghan L. Wilson^1*^, M.S., Thomas E. Strayer III^2^, M.S., Rebecca Davis^3^ M.S., Samantha M. Harden^1^, Ph.D

*** Correspondence:** Meghan L. Wilson [meghan13@vt.edu](mailto:meghan13@vt.edu)

# Supplementary Table: Example weekly process evaluation completed by Extension health educators

|  | **Week:** | **Week:** |
| --- | --- | --- |
| Date: |  |  |
| Agent ID: |  |  |
| Temperature: |  |  |
| # Of Participants Registered: |  |  |
| # Of Participants Present: |  |  |
| Scheduled Start Time: | _______AM or _______PM | _______AM or _______PM |
| Actual Start Time: | _______AM or _______PM | _______AM or _______PM |
| Were session materials  set-up prior to start time? | Circle: Yes No | Circle: Yes No |
| Was warm-up completed as  described in manual? | Circle: Yes No | Circle: Yes No |
| If no, what was different? |  |  |
| How many participants  completed the warm-up? |  |  |
| Was the group activity  completed as described  in manual? | Circle: Yes No | Circle: Yes No |
| If no, what changes were  made? |  |  |
| Did participants engage in  the group activity? | Circle: Yes No | Circle: Yes No |
| If no, what were they doing? |  |  |
| How did you cope with  participant behavior? |  |  |
| Did you use the correct count  for the 8 core exercises? | Circle: Yes No | Circle: Yes No |
| Did you complete all 8  exercises?  Wide Leg Squat  Leg Curl  Knee Extension  Side Hip Raise  Biceps Curls  Overhead Press  Seated Row  Toe Stand | Circle Yes or No and fill in # of  Yes or No 1)______ 2)_______  Yes or No 1)______ 2)_______  Yes or No 1)______ 2)_______  Yes or No 1)______ 2)_______  Yes or No 1)______ 2)_______  Yes or No 1)______ 2)_______  Yes or No 1)______ 2)_______  Yes or No 1)______ 2)_______ | repetitions for set 1 & set 2  Yes or No 1)______ 2)_______  Yes or No 1)______ 2)_______  Yes or No 1)______ 2)_______  Yes or No 1)______ 2)_______  Yes or No 1)______ 2)_______  Yes or No 1)______ 2)_______  Yes or No 1)______ 2)_______  Yes or No 1)______ 2)_______ |
| If no, what adaptations or  changes were made? |  |  |
| Was the cool-down  completed as described in the  manual? | Circle: Yes No | Circle: Yes No |
| If no, what changes were  made? |  |  |
| Did participants complete  the cool-down? | Circle: Yes No | Circle: Yes No |
| If no, what were they doing? |  |  |
| If no, how did you cope with  participant behavior? |  |  |
| Did you review homework  and reminders? | Circle: Yes No | Circle: Yes No |
| If no, why? |  |  |
| Did participants engage in  discussion or ask questions? | Circle: Yes No | Circle: Yes No |
| If so, were you able to  answer questions? | Circle: Yes No | Circle: Yes No |
| Overall, was the session  completed as intended? | Circle: Yes No | Circle: Yes No |

**
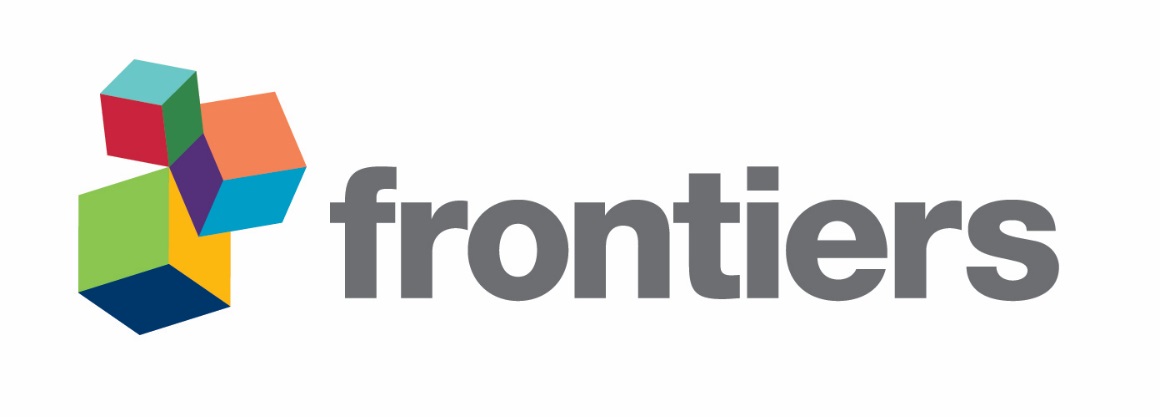
**
